# Phenotyping and Quantitative Trait Locus Analysis for the Limited Transpiration Trait in an Upper-Mid South Soybean Recombinant Inbred Line Population (“Jackson” × “KS4895”): High Throughput Aquaporin Inhibitor Screening

**DOI:** 10.3389/fpls.2021.779834

**Published:** 2022-01-20

**Authors:** Sayantan Sarkar, Avat Shekoofa, Angela McClure, Jason D. Gillman

**Affiliations:** ^1^Department of Plant Sciences, University of Tennessee, Knoxville, TN, United States; ^2^Plant Genetics Research Unit, USDA-ARS, University of Missouri, Columbia, MO, United States

**Keywords:** AgNO_3_, aquaporin inhibitor, genotyping, high throughput phenotyping, water conservation trait

## Abstract

Soybean is most often grown under rainfed conditions and negatively impacted by drought stress in the upper mid-south of the United States. Therefore, identification of drought-tolerance traits and their corresponding genetic components are required to minimize drought impacts on productivity. Limited transpiration (TR_lim_) under high vapor pressure deficit (VPD) is one trait that can help conserve soybean water-use during late-season drought. The main research objective was to evaluate a recombinant inbred line (RIL) population, from crossing two mid-south soybean lines (“Jackson” × “KS4895”), using a high-throughput technique with an aquaporin inhibitor, AgNO_3_, for the TR_lim_ trait. A secondary objective was to undertake a genetic marker/quantitative trait locus (QTL) genetic analysis using the AgNO_3_ phenotyping results. A set of 122 soybean genotypes (120-RILs and parents) were grown in controlled environments (32/25-d/n °C). The transpiration rate (TR) responses of derooted soybean shoots before and after application of AgNO_3_ were measured under 37°C and >3.0 kPa VPD. Then, the decrease in transpiration rate (DTR) for each genotype was determined. Based on DTR rate, a diverse group (slow, moderate, and high wilting) of 26 RILs were selected and tested for the whole plant TRs under varying levels of VPD (0.0–4.0 kPa) at 32 and 37°C. The phenotyping results showed that 88% of slow, 50% of moderate, and 11% of high wilting genotypes expressed the TR_lim_ trait at 32°C and 43, 10, and 0% at 37°C, respectively. Genetic mapping with the phenotypic data we collected revealed three QTL across two chromosomes, two associated with TR_lim_ traits and one associated with leaf temperature. Analysis of Gene Ontologies of genes within QTL regions identified several intriguing candidate genes, including one gene that when overexpressed had previously been shown to confer enhanced tolerance to abiotic stress. Collectively these results will inform and guide ongoing efforts to understand how to deploy genetic tolerance for drought stress.

## Introduction

Plant photosynthesis and transpirational rates are highly coupled (Sinclair, 2017). Most of the water lost by plants under stress is due to transpiration, which is linked to stomatal opening to allow CO_2_ diffusion (Devi and Reddy, 2018a; Shekoofa and Sinclair, 2020). Hence, water loss due to transpiration is linked to plant biomass growth and yield (Tanner and Sinclair, 1983; Blum, 2009). Low water availability reduces assimilate partitioning to reproductive sinks, and on a cellular level impairs cell growth and division (Blum, 2011; Sarkar, 2020; Bennett et al., 2021; Sarkar et al., 2021a). Water related stresses, such as direct drought stress and associated factors including high temperature and evaporative demand, reduce both transpiration and photosynthesis, resulting in reduced crop yield (Sinclair, 2017; Balota et al., 2021).

However, it is suggested that under extreme drought or high atmospheric vapor pressure deficit (VPD) conditions, restriction of stomatal conductance might increase photosynthetic return per unit of transpiration (Sinclair et al., 2005, 2010; Devi et al., 2009; Gholipoor et al., 2010; Carpentieri-Pipolo et al., 2012; Gaffney et al., 2015; Shekoofa et al., 2020). These traits result in restricted TR under high vapor pressure conditions, such that water is conserved in the soil and is available during subsequent drought periods. Thus, plants resort to drought avoidance mechanism such as limited transpiration (TR_lim_) and minimizing stomatal conductance (Kooyers, 2015; Basu et al., 2016; Devi et al., 2016). These studies also suggested that reduced TR and stomatal conductance are controlled by gene expression. Such mechanisms have been successfully studied in crop species, including: maize (*Zea Mays* L.) (Fletcher et al., 2007; Shekoofa et al., 2016), peanut (*Arachis hypogaea* L.) (Devi et al., 2009; Shekoofa et al., 2013, 2017), cotton (*Gossypium hirsutum* L.) (Devi and Reddy, 2018b; Shekoofa et al., 2020), sorghum (*Sorghum bicolor* L.) (Gholipoor et al., 2010; Choudhary et al., 2013), and soybean [*Glycine max* (L.) Merr.] (Bunce, 1981; Devi et al., 2014; Sarkar et al., 2021b). Of particular interest, soybean showed a decrease in stomatal conductance between VPD of 1.0 and 2.5 kPa, differing within genotypes (Bunce, 1981).

Decreases in transpiration rate and stomatal conductance are affected by leaf temperature (LT) (Gates, 1964; Blum, 2011). Gates (1964) argued convincingly that increase in LT can be lethal for plants and that transpiration is required to cool the leaves. The study also argues that even a slow rate of transpiration can dissipate enough heat from leaves to have a huge impact on photosynthesis and growth of plants. This implies that plants with slower rate of transpiration (with subsequently warmer leaves than plants with higher rates of transpiration) could be better drought-tolerant by conserving moisture during low water stress, which would be available later in the season when evaporative demand is higher. Under field conditions, drought-tolerant peanut genotypes (with partial stomata closure trait) displayed a downward LT slope with an increase in VPD during midday (1100 to 1400 EST) (Balota and Sarkar, 2020). Bai and Purcell (2018) observed an interaction between LT and slow and fast-wilting soyabean genotypes, where slow-wilting genotypes had a lower LT during water-deficit stress.

It is essential that any putative water saving trait should result in monetarily relevant yield benefit and should have genetic variability within the target crop species (Sadok and Sinclair, 2011). In fact, soybean genotype PI 416937 has been identified as expressing the TR_lim_ trait (i.e., slow-wilting) phenotype in the field under high VPD (>2.5 kPa), and this has been traced to low leaf hydraulic conductance (Sinclair et al., 2008). Sadok and Sinclair (2012) suggested that the low leaf hydraulic conductance in PI 416937 might be related to a unique population of aquaporins (AQPs) in its leaves. Further studies have shown that soybean genotypes expressing TR_lim_ trait are insensitive to aquaporin inhibitors, such as silver and zinc ions (Sadok and Sinclair, 2010, 2012; Devi et al., 2016).

In plants, AQPs occur in multiple isoforms in both plasmalemma and tonoplast membranes resulting in regulation of water flow in and out of cells. Physiological and molecular studies have identified AQPs as playing key roles in regulating hydraulic conductance in leaves and roots (Beaudette et al., 2007; Shekoofa and Sinclair, 2018; Sung et al., 2021). Therefore, the high-throughput phenotyping of structured soybean populations, along with molecular genotyping is needed for marker development. This type of research is essential for making the soybean genome sequence useful for breeding purposes, in particular for screening drought-tolerant soybean lines based on their sensitivity to aquaporin inhibitors.

In this work, we used a two-tiered screening method to identify soybean genotypes with the TR_lim_ trait. Our method is similar to the three-tiered approach by Sinclair et al. (2000). Direct measures of the transpiration response rate to increasing VPD are low-throughput, tedious, and require specialized equipment. Furthermore, the number of genotypes that can be directly phenotyped for the TR_lim_ trait is very limited. Thus, an indirect measurement of TR that may be less accurate but can allow characterization of a large number of genotypes would be helpful in breeding for the TR_lim_ trait (Choudhary and Sinclair, 2014). Choudhary and Sinclair (2014) suggested that one possibility is that the response in TR to feeding of chemical inhibitors to leaves or roots of plants might allow discrimination among genotypes under varying VPD levels. Appropriate parents have to be chosen for crossing when it comes to screening for drought tolerance traits. Previous studies have reported that soybean genotypes “KS4895” and “Jackson” differ in drought tolerance traits such as delayed wilting, ureide, and nitrogen concentration (Sinclair et al., 2007; Charlson et al., 2009; Hwang et al., 2013, 2015a,b; King et al., 2014). Therefore, the objectives of this study were to (a) evaluate 120 recombinant inbred lines (RILs; F3 and F5 derived from “KS4895” × “Jackson”) through a high-throughput phenotyping technique while measuring the RILs transpiration sensitivity rate to aquaporin inhibitor (AgNO_3_) under high VPD condition (initial/indirect screening for TR_lim_) (b) directly measure the TR of 26 selected RILs from objective “a” and categorize them for the TR_lim_ trait under high VPD and temperature (direct screening for TR_lim_), and finally (c) undertake genetics/quantitative trait loci (QTLs) analysis for the RIL population in objective “a” and associate the potential QTLs with the drought tolerance trait, i.e., TR_lim_.

## Materials and Methods

### Plant Materials and Study Site

The experiments were conducted at the University of Tennessee West Tennessee Research and Education Center (WTREC), in Jackson, TN between 2018 and 2020. A group of 122 soybean genotypes including 120 RILs and their parents “Jackson” and “KS4895” that were tested during the study (Table 1) were provided by Dr. Larry Purcell (Hwang et al., 2015a). Genotype “KS4895” (PI 595081) is a maturity group (MG) IV developed in Kansas (Schapaugh and Dille, 1998), and “Jackson” (PI 548657) is an MG VII genotype developed by the USDA-ARS in North Carolina (Johnson, 1958). Due to greenhouse space limitations, and to increase the accuracy during data collection since a large number of bottles were involved in each weighing process, only a subset of genotypes was tested with the silver nitrate test. Thus, the RILs were grown in six sets under controlled environments in a greenhouse at 33–35°C and 35–50% humidity during day, and 20–22°C and 45–60% humidity during the night. Each set was grown for 4 weeks and included both parents.

### Phenotyping and Phenotypic Traits

#### Experiment I

##### Indirect Measurement of Transpiration Under High Vapor Pressure Deficit: High Throughput Screening Using Aquaporins Inhibitor, AgNO_3_

Five soybean seeds were planted in 3-L pots filled with soil (commercially available Miracle-Gro potting mix) and inoculated with *Bradyrhizobium japonicum* (Verdesian Life Sciences, Cary, NC, United States). They were thinned to three plants per pot after 1 week in three-pot replicates per genotype. Plants were maintained in a well-watered condition, in 250 mL pots (i.e., pot capacity) during the initial growth pretreatment period. After approximately 4 weeks, the plants were ready for AgNO_3_ test. For the measurement of TR response to AQPs inhibitor, AgNO_3_, the technique explained by Sadok and Sinclair (2010), Shekoofa et al. (2013), and Devi et al. (2016) was followed.

The evening before the application of AgNO_3_, three replicate plants per soybean genotype were removed from the soil and derooted by cutting the base of the plant stem underwater using a sharp blade (Table 1). Then derooted soybean shoots were immediately placed in 150-mL Erlenmeyer flasks filled with deionized water. The shoots were kept in a dark laboratory room overnight for about 14 to 15 h with temperature maintained at 24°C. The following morning, the derooted shoots were transferred to another 150-mL Erlenmeyer flask containing fresh deionized water and sealed with Parafilm-“M” (Pechiney Plastic Packaging, Chicago, IL, United States) to avoid direct water evaporation from the flask. Then, in each set of experiment, the derooted shoots were moved to a greenhouse in which both temperature and VPD were high (Table 1).

**TABLE 1:** Soybean recombinant inbred lines (RILs) from crossing “Jackson” and “KS4895” evaluated in this study.

	RIL	Date	Temp (°C)	VPD (kPa)		RIL	Date	Temp (°C)	VPD (kPa)		RIL	Date	Temp (°C)	VPD (kPa)
**Set 1**	#38	05/23/2018	36–37	2.6–3.8	**Set 3**	#90	07/31/2018	35.5–38	2.5–4	**Set 6**	#7	04/10/2019	37–38	2.5–4.2
	#39	05/23/2018	36–37	2.6–3.8		#91	07/31/2018	35.5–38	2.5–4		#8	04/10/2019	37–38	2.5–4.2
	#40	05/23/2018	36–37	2.6–3.8		#92	07/31/2018	35.5–38	2.5–4		#12	04/10/2019	37–38	2.5–4.2
	#43	05/23/2018	36–37	2.6–3.8		#94	07/31/2018	35.5–38	2.5–4		#22	04/10/2019	37–38	2.5–4.2
	#44	05/23/2018	36–37	2.6–3.8		#95	07/31/2018	35.5–38	2.5–4		#24	04/10/2019	37–38	2.5–4.2
	#45	05/23/2018	36–37	2.6–3.8		#96	07/31/2018	35.5–38	2.5–4		#42	04/10/2019	37–38	2.5–4.2
	#49	05/23/2018	36–37	2.6–3.8		#97	07/31/2018	35.5–38	2.5–4		#46	04/10/2019	37–38	2.5–4.2
	#50	05/23/2018	36–37	2.6–3.8		#100	07/31/2018	35.5–38	2.5–4		#47	04/10/2019	37–38	2.5–4.2
	#51	05/23/2018	36–37	2.6–3.8		#102	07/31/2018	35.5–38	2.5–4		#53	04/10/2019	37–38	2.5–4.2
	#52	05/23/2018	36–37	2.6–3.8		#104	07/31/2018	35.5–38	2.5–4		#61	04/10/2019	37–38	2.5–4.2
	#54	05/23/2018	36–37	2.6–3.8		#107	07/31/2018	35.5–38	2.5–4		#68	04/10/2019	37–38	2.5–4.2
	#55	05/23/2018	36–37	2.6–3.8		#108	07/31/2018	35.5–38	2.5–4		#72	04/10/2019	37–38	2.5–4.2
	#56	05/23/2018	36–37	2.6–3.8		#109	07/31/2018	35.5–38	2.5–4		#77	04/10/2019	37–38	2.5–4.2
	#57	05/23/2018	36–37	2.6–3.8	**Set 4**	#113	08/07/2018	37–38	3–4.2		#78	04/10/2019	37–38	2.5–4.2
	#58	05/23/2018	36–37	2.6–3.8		#115	08/07/2018	37–38	3–4.2		#80	04/10/2019	37–38	2.5–4.2
	#59	05/23/2018	36–37	2.6–3.8		#117	08/07/2018	37–38	3–4.2		#86	04/10/2019	37–38	2.5–4.2
	#62	05/23/2018	36–37	2.6–3.8		#120	08/07/2018	37–38	3–4.2		#88	04/10/2019	37–38	2.5–4.2
	#64	05/23/2018	36–37	2.6–3.8		#121	08/07/2018	37–38	3–4.2		#93	04/10/2019	37–38	2.5–4.2
	#66	05/23/2018	36–37	2.6–3.8		#125	08/07/2018	37–38	3–4.2		#98	04/10/2019	37–38	2.5–4.2
	#70	05/23/2018	36–37	2.6–3.8		#127	08/07/2018	37–38	3–4.2		#106	04/10/2019	37–38	2.5–4.2
**Set 2**	#2	07/05/2018	36–37	2.5–3.5		#129	08/07/2018	37–38	3–4.2		#124	04/10/2019	37–38	2.5–4.2
	#3	07/05/2018	36–37	2.5–3.5		#131	08/07/2018	37–38	3–4.2		#128	04/10/2019	37–38	2.5–4.2
	#4	07/05/2018	36–37	2.5–3.5		#133	08/07/2018	37–38	3–4.2		#130	04/10/2019	37–38	2.5–4.2
	#9	07/05/2018	36–37	2.5–3.5		#134	08/07/2018	37–38	3–4.2		#135	04/10/2019	37–38	2.5–4.2
	#11	07/05/2018	36–37	2.5–3.5		#136	08/07/2018	37–38	3–4.2		#138	04/10/2019	37–38	2.5–4.2
	#13	07/05/2018	36–37	2.5–3.5		#137	08/07/2018	37–38	3–4.2		#143	04/10/2019	37–38	2.5–4.2
	#14	07/05/2018	36–37	2.5–3.5		#139	08/07/2018	37–38	3–4.2		#144	04/10/2019	37–38	2.5–4.2
	#16	07/05/2018	36–37	2.5–3.5		#140	08/07/2018	37–38	3–4.2		#147	04/10/2019	37–38	2.5–4.2
	#17	07/05/2018	36–37	2.5–3.5		#141	08/07/2018	37–38	3–4.2		#151	04/10/2019	37–38	2.5–4.2
	#18	07/05/2018	36–37	2.5–3.5		#142	08/07/2018	37–38	3–4.2		#152	04/10/2019	37–38	2.5–4.2
	#20	07/05/2018	36–37	2.5–3.5		#149	08/07/2018	37–38	3–4.2					
	#23	07/05/2018	36–37	2.5–3.5		#153	08/07/2018	37–38	3–4.2					
	#26	07/05/2018	36–37	2.5–3.5		#154	08/07/2018	37–38	3–4.2					
	#30	07/05/2018	36–37	2.5–3.5	**Set 5**	#155	08/14/2018	37–38	3–4.3					
	#31	07/05/2018	36–37	2.5–3.5		#156	08/14/2018	37–38	3–4.3					
	#34	07/05/2018	36–37	2.5–3.5	#157	08/14/2018	37–38	3–4.3				
	#35	07/05/2018	36–37	2.5–3.5	#160	08/14/2018	37–38	3–4.3				
	#36	07/05/2018	36–37	2.5–3.5	#161	08/14/2018	37–38	3–4.3				
	#71	07/05/2018	35.5–38	2.5–4	#162	08/14/2018	37–38	3–4.3				
	#73	07/05/2018	35.5–38	2.5–4	#164	08/14/2018	37–38	3–4.3				
	#74	07/05/2018	35.5–38	2.5–4	#165	08/14/2018	37–38	3–4.3				
	#79	07/05/2018	35.5–38	2.5–4	#166	08/14/2018	37–38	3–4.3				
	#81	07/05/2018	35.5–38	2.5–4	#168	08/14/2018	37–38	3–4.3				
	#87	07/05/2018	35.5–38	2.5–4	#169	08/14/2018	37–38	3–4.3				
	#89	07/05/2018	35.5–38	2.5–4	#170	08/14/2018	37–38	3–4.3				

*The plants were grown at 33–35°C and 35–50% humidity during day, and 20–22°C and 45–60% humidity during night. The date in this table presents the data collection date per each set for “experiment 1.” The temperature (temp) and vapor pressure deficit (VPD) are the conditions that the plants were subjected to on the day of data collection. Along with the RILs both parents were tested in each set of data collection.*

Water-cooled lamps provided the photosynthetic photon flux density, which was 600 μmol m^–2^ s^–1^ at plant level (Mullen and Koller, 1988; Devi et al., 2016). The derooted shoots were allowed to acclimatize for 60 min and then the flasks plus derooted shoots were weighed (TR H_2_O initial). After another 60 min, the flasks plus derooted shoots were reweighed (TR H_2_O final), and the difference between the two weights divided by the time interval was used to calculate the TR in water (TR_H2O_). Following the second weighing of the derooted shoots in water, the individual shoots were quickly transferred to dark-brown, 30-mL glass bottles for exposure to the solution of 200 μM, AgNO_3_ aquaporin inhibitor (Sadok and Sinclair, 2010). The AgNO_3_ solution was freshly prepared on the day before each set of experiment. The AgNO_3_ solution concentration of 200 μM was selected because this concentration was the lowest concentration resulting in maximum transpiration decrease (Sadok and Sinclair, 2010). The derooted shoots were allowed to take up the AgNO_3_ solution for 60 min by which time TR of the shoots had again reached a constant value (Sadok and Sinclair, 2010).

After the 60 min period, all bottles were weighed to get an initial weight for the transpiration measurement following exposure to AgNO_3_ (TR AgNO_3_ initial). After 180 min, each bottle was again weighed (TR AgNO_3_ final). Measurements of TR for each shoot were generally completed in about 240 min after AgNO_3_ treatment. The TR following exposure to silver (TR_AgNO3_) was calculated based on the difference between these two weights divided by the time interval. The difference between TR_H2O_ and TR_AgNO3_ were used to quantify decrease in TR using various arithmetic combinations (Table 2).

**TABLE 2:** Various transpiration rate parameters and its derivatives used for evaluation of soybean genotypes, “experiment 1.”

Transpiration rate parameter	Full form	Formula
**TR_H2O_**	Transpiration rate in water	$\frac{\left(\mathrm{TR}\,\mathrm{H2O}\,\mathrm{initial}\right)-\left(\mathrm{TR}\,\mathrm{H2O}\,\mathrm{final}\right)}{\mathrm{Time}\,\mathrm{interval}}$
**TR_AgNO3_**	Transpiration rate following the aquaporin inhibitor (AgNO_3_) exposure	$\frac{\left({\text{TRAgNO}}_{3}\,\mathrm{initial}\right)-\left({\text{TRAgNO}}_{3}\,\mathrm{final}\right)}{\mathrm{Time}\,\mathrm{interval}}$
**DTR**	Decrease in transpiration rate	$\frac{{\text{TRH}}_{2}\,O-\mathrm{TR}\,{\text{AgNO}}_{3}}{{\text{TRH}}_{2}\,\text{O}}\times 100$
**RTR**	Ratio of transpiration rate	$\frac{{\text{TRH}}_{2}\,\text{O}}{{\text{TRAgNO}}_{3}}$
**NDTR**	Normalized decrease in transpiration rate	$\frac{{\text{TRH}}_{2}\,O-\mathrm{TR}\,{\text{AgNO}}_{3}}{{\text{TRH}}_{2}\,\text{O}+\mathrm{TR}\,{\text{AgNO}}_{3}}$
**RDTR_*J*_**	Relative decrease in transpiration rate by “Jackson”	$\frac{\text{DTR}}{\mathrm{DTR}\,\mathrm{of}\,\mathrm{Jackson}}$
**RDTR_K_**	Relative decrease in transpiration rate by “KS4895”	$\frac{\text{DTR}}{\mathrm{DTR}\mathrm{of}“\mathrm{KS4895}{}^{\prime \prime }}$
**RRTR_J_**	Relative ratio of transpiration rate by “Jackson”	$\frac{\text{RTR}}{\mathrm{RTR}\mathrm{of}“\text{Jackson}{}^{\prime \prime }}$
**RRTR_K_**	Relative ratio of transpiration rate by “KS4895”	$\frac{\text{RTR}}{\mathrm{RTR}\mathrm{of}“\mathrm{KS4895}{}^{\prime \prime }}$
**RNDTR_J_**	Relative normalized decrease in transpiration rate by “Jackson”	$\frac{\text{NDTR}}{\mathrm{NDTR}\mathrm{of}“\text{Jackson}{}^{\prime \prime }}$
**RNDTR_K_**	Relative normalized decrease in transpiration rate by “KS4895”	$\frac{\text{NDTR}}{\mathrm{NDTR}\mathrm{of}“\mathrm{KS4895}{}^{\prime \prime }}$

##### Leaf Temperature

In each set, LT was measured for all tested derooted shoots using a FLIR C3 Thermal Camera with WiFi (Teledyne FLIR LLC, Wilsonville, OR, United States) between 1300 and 1500 CST. In each set of experiment, the corresponding thermal images were taken 1 m above the plant at a resolution of 0.9 cm per pixel for three replications of derooted shoots. The temperature at the central point of a fully expanded trifoliate leaf was obtained for each of the three plants from a thermal image using the camera software. The thermal data were collected after the last stage of exposure to AgNO_3_ when the final weighing was done. These data sets were used for calculating the average LT for each genotype.

##### Statistical Analysis

A mixed linear model was applied to TR response data using the Fit Model function of JMP 14 (SAS Institute) using an input of 120 RIL lines (and two parental lines), for which we had both genotypic and phenotypic information. For mixed linear models, three factors were included: (1) genotype; (2) experiment; and (3) replication within each experiment. Only genotype was considered fixed, whereas other factors were considered random, and replication was nested within experiment. Principle component analysis (PCA) was run to create a PCA biplot of phenotypic measurements. Pearson’s correlation matrix was used to measure correlation among phenotypes. Polynomial regression was used to explain the variation of relative decrease in TR due to LT.

##### Broad Sense Heritability

Broad sense heritability (H^2^) of all traits was calculated as the ratio of genotypic variance ($\sigma_{G}^{2})$ by phenotypic variance ($\sigma_{P}^{2}$) (Phansak et al., 2016). Variance was calculated as the ratio of total sum of squares (TSS) to population size (*n*). The heritability data have been included in Supplementary File 1.


$$H^{2}=\frac{\sigma_{G}^{2}}{\sigma_{G}^{2}+\frac{\sigma_{G}^{2}\,\sigma_{E}^{2}}{E}+\frac{\sigma_{E}^{2}}{E\,R}}$$

where $\sigma_{P}^{2}=\sigma_{G}^{2}+\frac{\sigma_{G}^{2}\,\sigma_{E}^{2}}{E}+\frac{\sigma_{E}^{2}}{E\,R}$; $o_{\text{E}}^{2},\mathrm{environmental}\,\mathrm{variance};E,\mathrm{number}\,\mathrm{of}\,\mathrm{environments};$R, number of replications.

#### Experiment II

##### Direct Measurement of Transpiration Under High Vapor Pressure Deficit

Twenty-six soybean genotypes (24 RILs and both parents), based on RDTR_K_ and RNDTR_K_ (from the indirect measurement of transpiration, “Experiment I”), were selected to be tested for the direct TR measurement under varying levels of VPD (0.5 to 4.0 kPa). Soybean genotypes that had RDTR_K_ and RNDTR_K_ values from 0.00 to 0.50 were considered slow wilting, 0.51 to 1.00 were considered moderate wilting, and above 1.00 were considered as fast wilting. The plants were grown in pots constructed from polyvinyl chloride pipe (100-mm diameter and 200-mm long). The bottom of each pot was fitted with a flat end cap, in which a small hole was drilled to allow drainage of excess water. A toilet flange was attached to the top of the pot to allow easy attachment of a VPD chamber during measurements (Fletcher et al., 2007; Shekoofa et al., 2013). The pots were filled with commercially available Miracle-Gro potting mix. Five seeds per pot were sown and inoculated with *B. japonicum* (Verdesian Life Sciences, Cary, NC, United States). After 1 week, the plants were thinned to one plant per pot. Plants were fertilized with 200 mL of 0.075% V/V liquid fertilizer (0-10-10, N-P_2_O_5_-K_2_O, GH Inc., Sebastopol, CA, United States) at planting and again at 14 and 24 days after planting (DAP). Plants were grown for 28 days under well-watered conditions, with greenhouse temperatures regulated at 33°C day/26°C night. After approximately 4 weeks, four replicate plants of each genotype were selected and moved into a walk-in growth chamber and enclosed in individual humidity-regulated chambers (21 L). As there were 12 individual chambers, this meant that the genotypes had to be measured in batches that included three genotypes in each batch.

Measurements of transpiration response to VPD were carried out at 32 and 37°C. Each individual VPD chamber (21 L) was fitted with a 12-V, 80-mm-diameter cooling fan (Masscool) to continuously stir the air inside the chamber. Stirring of the air helped to maintain plant temperature near ambient air temperature within the VPD chamber. A humidity/temperature data logger (Lascar Electronics) was mounted through the sidewall of each container to monitor the environmental conditions of the chamber. The plants were subjected to three VPD levels: low (0–1.5 kPa), medium (1.5–2.5 kPa), and high (2.5–4 kPa), on two consecutive days for each temperature (i.e., 32 and 37°C). The humidity in the VPD chambers was obtained by adjusting the airflow rate through each chamber; in the case of the highest VPD treatment (2.5–4 kPa), the air was also initially flowed through a column of silica gel to dry the input air (Fletcher et al., 2007).

The observations of all plants for each genotype were combined for a two-segment linear regression analysis (Prism 8.0, GraphPad, Software Inc., San Diego, CA, United States) of TR versus varying VPD at both levels 32 and 37°C temperatures.

##### Statistical Analysis

The outputs of a successful regression fit to the two-segment model were the coefficients defining two intersecting linear regressions and the VPD value at the breakpoint (BP) between the two linear segments:


If VPD<BP,TR=Slope⁢ 1⁢(VPD)+Intercept⁢ 1.

If VPD>BP,TR=Slope⁢ 2⁢(VPD)+Intercept⁢ 2.

The slopes of the two linear regressions (Slopes 1 and 2) were statistically compared to determine whether they differed significantly (*p* < 0.05). If the slopes differed, the double-linear regression was retained. When the slopes were not significantly different, a simple linear regression was applied to all the data (Devi et al., 2010; Shekoofa et al., 2020).

### Genotyping and Quantitative Trait Locus Discovery

#### Genetic Map Construction

DNA was isolated from lyophilized leaf tissue for 118 out of 120 RIL lines, as well as the parental lines “KS4895” and “Jackson” using a Promega AS1600 kit (Promega, Madison, WI, United States) using a Maxwell RSC instrument (Promega). Two genotypes did not germinate and the leaf tissue could not be collected. Genotypes for genetic map creation and QTL detection were obtained using the SoySNP6k iSelect BeadChip (Song et al., 2020), an Illumina Infinium^®^ HD array. Genotypes were called using the iScan and Genome Studio software (Illumina, San Diego, CA, United States) by Dr. Qijian Song and Chuck Quigley of the USDA-ARS. Genotypic results were imported into TASSEL 5 (Bradbury et al., 2007), where nonsegregating markers were removed, missing genotypes were imputed using LinkImpute (Money et al., 2015), functionality incorporated into TASSEL 5 (settings = 30 High LD sites, 10 nearest, 10,000,000 maximum distance), and imputed genotypes converted to ABH format (AA = “Jackson” allele homozygote, AB = heterozygote, BB = “KS4895” allele homozygote) using the ABH Genotype add-in. Data were then error-corrected using the R package ABHGenotypeR (Reuscher and Furuta, 2016) with settings maxHapLength = 5 used throughout. This resulted in 2,184 total genetic markers in the final genetic map. Genotypic data were then merged with phenotypic information and imported in the QTL package in R (Broman and Sen, 2009). Distance between genetic markers was estimated using the est.map function with settings typical of an F3 population (error.prob = 0.01, overall genotypes ratios were AA = 33.0%, AB = 33.8%, and BB = 33.3%). Markers which introduced large gaps or increased genetic map length were identified using the droponemarker^1^ command and manually removed if they reduced the size of the overall genetic mapping significantly.

Summary information on the genetic map is located Supplementary File 2 and the complete R/QTL file containing both genotypic and least square means (LSM) phenotypic data is located in Supplementary File 3.

#### Quantitative Trait Locus Analysis

Genetic mapping was performed within the R/QTL program (Broman and Sen, 2009) using two different QTL detection methods: standard interval mapping (SIM) and composite interval mapping (CIM) using the computer package R/QTL (Broman and Sen, 2009). Lod thresholds were determined from 1,000 permutation testing for each trait. Allelic effects were estimated after using “sim.geno” function (16 draws and an error probability of 0.01). QTL were used to build an additive model, which was fitted, refined, and then refitted using the functions “makeqtl,” “refineqtl,” and “fitqtl.” Results were extremely concordant between SIM and CIM; for brevity and clarity only CIM results are presented in this study.

#### Candidate Gene Analysis of Quantitative Trait Locus Regions

Genes present in QTL regions were examined to identify potential candidate genes for the TR_lim_ traits using the *G. max* genome assembly version Glyma.Wm82.a2.1 (Schmutz et al., 2010). Annotations were downloaded.^2^

We examined three detected QTL windows (Supplementary Table 1) to investigate Gene Ontologies (GOs) terms for biological process, cellular component, and molecular function. Genes were termed as candidates if they had biological process GO terms associated with: abscisic acid, water transport, root development, leaf senescence, jasmonic acid, heat acclimation, stomata, and/or salicylic acid (Schulze, 1986; Jackson et al., 2000; Schmutz et al., 2010; Khan et al., 2012; Jarzyniak and Jasinski, 2014; Sah et al., 2016).

## Results

### Phenotyping and Phenotypic Traits

#### Indirect Measurement of Transpiration Under High Vapor Pressure Deficit: High Throughput Screening Using Aquaporins Inhibitor, AgNO_3_

**FIGURE 1:**
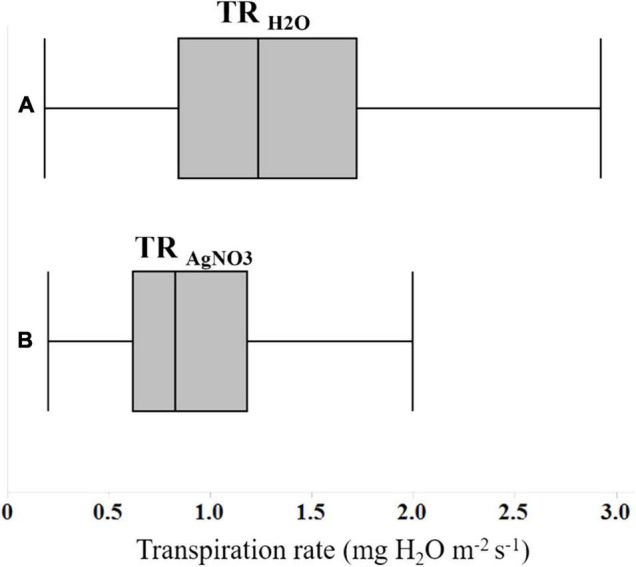
Box and whisker plots showing the differences in transpiration rates of 122 soybean genotypes **(A)** in water, and **(B)** following the exposure to an aquaporin inhibitor (AgNO_3_).

**FIGURE 2:**
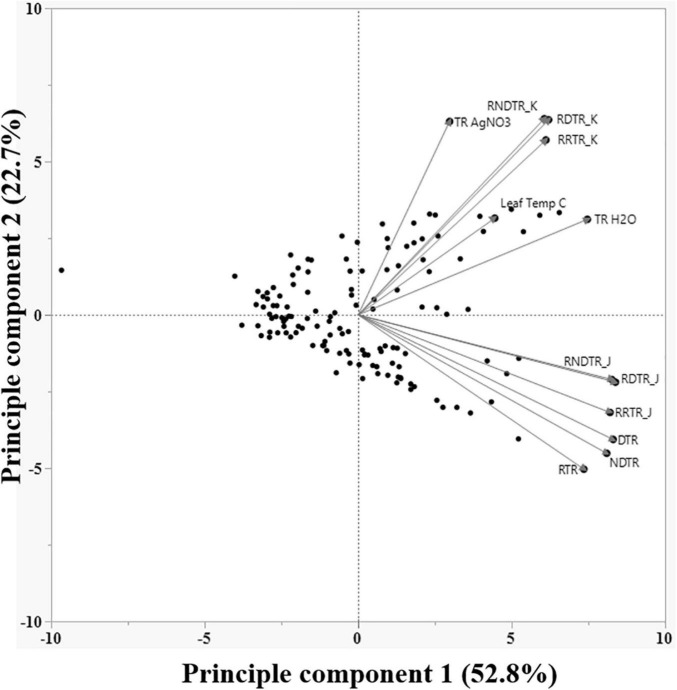
Principle component biplot of various phenotypic traits of all 122 soybean genotypes evaluated in this work. Traits farther from the center have higher variance, and higher the angle between traits lower the correlation.

**FIGURE 3:**
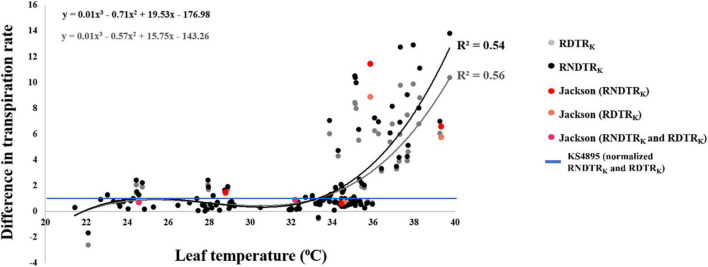
Regression curves of relative difference in transpiration rate response by “KS4895” (RDTR_K_) and relative normalized difference in transpiration rate by “KS4895” (RNDTR_K_) over the leaf temperature for 122 soybean genotypes.

Three primary traits (TR_H2O_, TR_AgNO3_, and LT) and nine secondary traits (DTR, RTR, NDTR, RDTR_J_, RDTR_K_, RRTR_J_, RRTR_K_, RNDTR_J_, and RNDTR_K_) were phenotyped in the experiment for all tested genotypes (Table 2). Phenotypic values for the three primary traits followed a normal distribution for LT and an approximately normal distribution for TR_H2O_ and TR_AgNO3_ (Table 3). Mean, upper 95% mean, and lower 95% mean of TR_H2O_ were higher than TR_AgNO3_ (1.32, 0.1.43, 1.21 for TR_H2O_ compared with 0.88, 0.95, 0.81 for TR_AgNO3_) (Table 3 and Figure 1). Across RILs genotypes, #162, #89, #97, #74, #16, #31, #161, #30, #168, #71, #26, and #137 had very low DTR (0–5%) and NDTR (0–0.02) whereas genotypes #52, #64, #17, #157, #91, #23, #34, #136, #73, #45, and #57 had low DTR (5–10%) and NDTR (0.03–0.05) (Supplementary Figure 1). However, the LT had better correlation with RDTR_K_ (*r* = 0.52, *p* < 0.0001) and RNDTR_K_ (*r* = 0.49, *p* < 0.0001) as compared with DTR (*r* = 0.39, *p* < 0.0001) and NDTR (*r* = 0.38, *p* < 0.0001) (Table 4 and Figure 2). Furthermore, the analysis of data by a polynomial regression showed that the variation in RDTR_K_ and RNDTR_K_ could be explained by LT (*R*^2^ = 0.54 and 0.56) (Figure 3). Therefore, RDTR_K_ and RNDTR_K_ were used to select a diversity of genotypes for further screening.

**TABLE 3:** Summary of the phenotypic values distribution for greenhouse “experiment 1.”

Category	Least Sq mean LT	Least Sq mean TR_AgNO3_	Least Sq mean TR_H2O_
**Mean**	32.51	0.88	1.32
**SD**	1.25	0.38	0.60
**SEM**	0.11	0.03	0.05
**Upper 95% mean**	32.73	0.95	1.43
**Lower 95% mean**	32.28	0.81	1.21
***N***	122	122	122
**Variance**	1.56	0.14	0.36
**Skewness**	0.27	0.66	0.52
**Kurtosis**	0.57	−0.30	−0.14
**CV**	3.84	42.92	45.34
**Jackson (RIL parent)**	33.33 ± 0.86	0.94 ± 0.06	1.4 ± 0.1
**KS4895 (RIL parent)**	32.04 ± 0.86	0.8 ± 0.06	1.23 ± 0.1

**TABLE 4:** Pearson’s correlation matrix of all direct and traits measured in this study.

	Leaf temp (°C)	TR_H2O_	TR_AgNO3_	DTR	RTR	NDTR	RDTR_J_	RDTR_K_	RRTR_J_	RRTR_K_	RNDTR_J_	RNDTR_K_
**Leaf temp (°C)**	1.00	***	***	***	***	***	***	***	**	***	**	***
**TR_H2O_**	0.47	1.00	***	***	***	***	***	***	***	***	***	***
**TR_AgNO3_**	0.32	0.79	1.00	ns	***	**	***	***	**	***	***	***
**DTR**	0.39	0.50	−0.06	1.00	***	**	***	***	***	***	***	***
**RTR**	0.34	0.44	−0.16	0.91	1.00	**	***	***	***	***	***	***
**NDTR**	0.38	0.49	−0.10	0.98	0.96	1.00	***	***	***	***	***	***
**RDTR_J_**	0.14	0.57	0.20	0.79	0.59	0.71	1.00	***	***	***	***	***
**RDTR_K_**	0.52	0.53	0.38	0.32	0.18	0.28	0.36	1.00	***	***	***	***
**RRTR_J_**	0.10	0.58	0.10	0.76	0.79	0.79	0.81	0.28	1.00	***	***	***
**RRTR_K_**	0.24	0.52	0.41	0.23	0.13	0.19	0.47	0.85	0.43	1.00	***	***
**RNDTR_J_**	0.10	0.59	0.19	0.76	0.62	0.72	0.98	0.35	0.88	0.50	1.00	***
**RNDTR_K_**	0.49	0.52	0.37	0.30	0.18	0.26	0.33	1.00	0.28	0.85	0.33	1.00

Bottom left is the coefficient of correlation, top right half is significance level. Significance levels: ***P < 0.0001; **P < 0.01; ns, non-significant at P > 0.05.

#### Direct Measurement of Transpiration Under High Vapor Pressure Deficit

**FIGURE 4:**
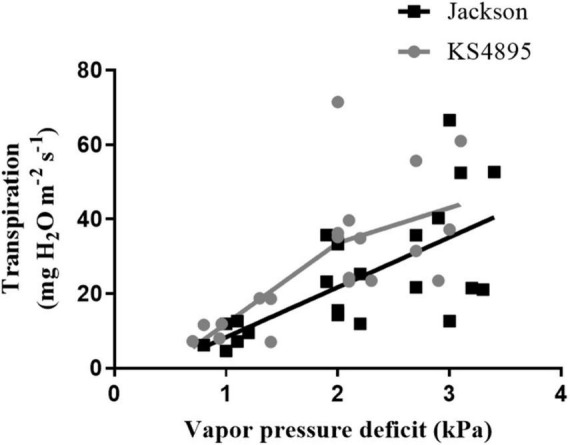
Transpiration rate response of two soybean genotypes (“Jackson” and “KS4895”) over different levels of vapor pressure deficit at 32°C.

Based on RDTR_K_ and RNDTR_K_ values, 26 soybean genotypes were divided into three levels. Values from 0.00 to 0.50 were considered as “slow wilting,” 0.51 to 1.00 as “moderate wilting,” and above 1.00 “fast wilting.” Genotypes exhibited a decrease in TR slope over increasing VPD, indicating the expression of the TR_lim_ trait, and those with constant linear slope exhibited no decrease in TR under high VPD, indicating the lack of the TR_lim_ trait. For example, between two parents “KS4895” expressed the TR_lim_ trait and “Jackson” did not (Figure 4). For those genotypes that expressed the TR_lim_ trait, the point on *x*-axis where the slope changed was considered as the VPD breakpoint. Within slow wilting genotypes, 6 out of 7 were found to express the TR_lim_ trait (i.e., VPD breakpoint) at high VPD under 32°C (Table 5). Among genotypes in moderate wilting category, 5 out of 10, and in high wilting only 1 out of 9 expressed the TR_lim_ trait at 32°C. Whereas, at 37°C, the expression of TR_lim_ trait was found to be limited only to 3 out of 7 in slow wilting, 1 out of 10 in moderate wilting, and none among fast wilting genotypes.

**TABLE 5:** Vapor pressure deficit (VPD) breakpoints of 26 soybean genotypes at 32 and 37°C.

	Genotypes	RDTR_K_	RNDTR_K_	VPD breakpoint at	
				32°C	37°C
**Slow wilting**	***Low-rate RDTR_K_ and RNDTR_K_***				
	#16	0.14	0.13	1.7	2.10
	#91	0.24	0.22	2.7	Linear
	#23	0.29	0.27	1.4	3.34
	#73	0.30	0.27	2.9	1.96
	#22	0.35	0.26	1.3	Linear
	#79	0.43	0.40	2.8	Linear
	#151	0.49	0.38	Linear	Linear
**Moderate wilting**	***Mid-rate RDTR_K_ and RNDTR_K_***				
	#87	0.71	0.67	3.0	2.39
	#102	0.72	0.68	Linear	Linear
	#24	0.75	0.66	2.2	Linear
	#168	0.78	0.78	Linear	Linear
	#55	0.79	0.76	Linear	Linear
	#93	0.79	0.71	2.1	Linear
	#14	0.86	0.84	Linear	Linear
	#56	0.92	0.91	Linear	Linear
	#147	0.93	0.90	2.9	Linear
	“KS4895”	1.00	1.00	2.0	Linear
**Fast wilting**	***High-rate RDTR_K_ and RNDTR_K_***				
	#35	1.07	1.08	2.8	Linear
	#152	1.08	1.12	Linear	Linear
	#51	1.42	1.52	Linear	Linear
	#108	1.51	1.64	Linear	Linear
	#3	1.81	2.02	Linear	Linear
	“Jackson”	3.05	3.61	Linear	Linear
	#134	3.28	3.49	Linear	Linear
	#142	6.24	7.25	Linear	Linear
	#139	9.77	12.73	Linear	Linear

*These genotypes have been selected based on “experiment 1” results. The wilting categories (slow, moderate, and fast) were determined based on the RDTR_K_ and RNDTR_K_ values. Values from 0.0 to 0.50 were considered slow wilting, 0.51 to 1.0 were considered moderate wilting, and above 1.0 were considered as fast wilting. Both parents were included with the RILs.*

### Genotyping and Quantitative Trait Locus Discovery

A genetic map was created from genotypic data obtained using the SoySNP6K Illumina array (Akond et al., 2013). The final genetic map was composed of 2,181 genetic markers (average 109/chromosome) encompassing a total of 2,856.4 cm (each chromosome was 142.8 cm on average) (Supplementary File 2).

**FIGURE 5:**
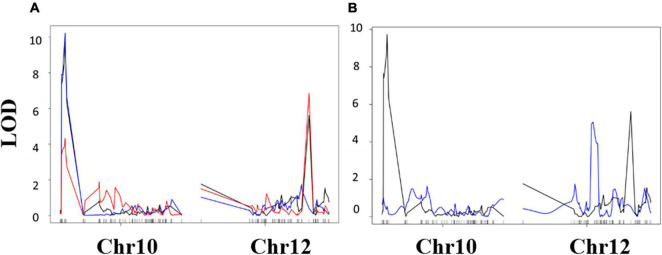
Composite interval mapping results. **(A)** Transpiration rate response (mg H_2_O m^–2^ s^–1^) QTL mapping results. Black indicates TR_H2O_, blue indicates TR_AgNO3_, and red indicates TR_H2O_ – TR_AgNO3_. **(B)** Leaf temperature (LT) QTL mapping results, black indicates TR_H2O_ results, and blue indicates LT results. Vertical axis is logarithm of odds (LOD) score. Horizontal axis is markers along chromosomes 10 and 12, ascending in genetic position from left to right.

Quantitative trait locus analysis was performed for four drought-related traits using LSM calculated from phenotypic measurements taken during our greenhouse experiments (Table 6). Three QTLs were identified in total (Tables 6, 7), two QTLs for the TR traits (qTR_Gm10_1 and qTR_Gm12_1), and one associated with LT (qLT_Gm12_1) (Figure 5).

**TABLE 6:** Quantitative trait locus mapping results.

Trait	QTL name	Coincident QTL	QTL peak	QTL interval	df	Type III SS	LOD	%var	*P*-value (Chi2)	*P*-value (F)	Allelic effect^1^
TR-H_2_O	qTR_Gm10_1	qSV_Gm10^2^	Gm10@6.6 cM	2–14 cM	2	10.20	9.26	27.59	0	1.36E-09	−0.63 mg H_2_O m2^–1^ s^–1^
	qTR_Gm12_1	qSV_Gm12^2^	Gm12@138.0 cM	136–142 cM	2	5.29	5.21	14.29	0	1.03E-05	0.27 mg H_2_O m2^–1^ s^–1^
TR-AgNO_3_	q TR_10_1	qSV_Gm10^2^	Gm10@6.6 cM	4–19 cM	2	2.57	7.57	25.58	2.69E-08	4.20E-08	−0.35 mg H_2_O m2^–1^ s^–1^
TR H_2_O – AgNO_3_	qTR_Gm10_1	qSV_Gm10^2^	Gm10@6.6 cM	0–18 cM	2	2.48	4.45	13.75	0	5.43E-05	−0.28 mg H_2_O m2^–1^ s^–1^
	qTR_Gm12_1	qSV_Gm12^2^	Gm12@138.0 cM	136–142 cM	2	3.37	5.87	18.64	0	2.40E-06	0.25 mg H_2_O m2^–1^ s^–1^
LT (°C)	qLT_Gm12_1	-	Gm12@91.0 cM	81–98 cM	2	30.09	4.80	17.10	1.57E-05	2.08E-05	−0.84°C

*^1^Estimated allelic effect of homozygous Jackson >> KS4895.*

*^2^qSV_Gm10 and qSV_Gm10 QTLs were previously reported in Carpentieri-Pipolo et al. (2012).*

**TABLE 7:** Candidate gene summary for three QTL regions.

Trait	QTL	QTL interval	Physical QTL interval (Gmax.W82.a2.1)	Total # genes in region	Candidate genes in region^1^
**TR H_2_O; TRAgNO_3_; (TR H_2_O – TRAgNO_3_)**	qTR_Gm10_1	4–19 cM	Gm10:116991–1903082	81	8
**TR H_2_O; (TR H_2_O – TRAgNO_3_)**	qTR_Gm12_1	136–142 cM	Gm12:35946078–36388059	55	8
**LT (°C)**	qLT_Gm12_1	81–98 cM	Gm12:6971475–11867391	312	29

*^1^Candidate genes were determined based on presence of GO terms associated with abscisic acid, water transport, root development, leaf senescence, jasmonic acid, stomata, and/or salicylic acid.*

Furthermore, the effect of each QTL was estimated (Table 6). Alleles from “KS4895” for the qTR_Gm10_1 QTL were associated with reduced transpiration relative to “Jackson” (−0.63 mg H_2_O m2^–1^ s^–1^). In contrast, alleles from “Jackson” for the qTR_Gm12_1 QTL were associated with reduced transpiration relative to “KS4895” (−0.27 mg H_2_O m2^–1^s^–1^). The lowest TR (and presumably most water saving) lines had a combination of QTL from two different parents: qTR_Gm10_1 for “KS4895” allele and qTR_Gm12_1 for the “Jackson” allele (Table 7). Lines which inherited alleles from “KS4895” for qLT_Gm12_1, which was distinct from the TR QTL, had lower leaf temperatures (0.84°C lower on average).

### Identification of Candidate Genes From Quantitative Trait Locus Regions

For genes within three detected QTL windows in this work (Table 6) using the “Williams 82” genome assembly 2 (Glyma.W82.a2.1), we examined gene ontologies (biological process, cellular component, and molecular function). Genes were termed as candidate genes (Table 7 and Supplementary Table 1) for the TR_lim_ traits if they had biological process GO terms associated with any of the following terms: abscisic acid, water transport, root development, leaf senescence, jasmonic acid, heat acclimation, stomata, and/or salicylic acid. It should be noted that very few genes in soybean have direct experimental or empirical evidence for gene function, and in large part gene annotations are inferred from homologs identified by BLAST searches using genomic assemblies from other species (typically Arabidopsis).

Within the qTR_Gm10_1 region (Gm10:116991–1903082; Table 7), we identified eight candidate genes (Table 7 and Supplementary Table 1). These included three ubiquitin signaling associated genes (Glyma.10G018800, Glyma.10G019000, Glyma.10G019500, and Glyma.10G021500), a glycoside hydrolase/polygalacturonase gene (Glyma.10G016100), an abiotic stress-associated transcription factor (Glyma.10G016500) and two genes of unknown function annotated as involved in root hair cell differentiation (Glyma.10G016600 and Glyma.10G016700). One very intriguing candidate gene is Glyma.10G016600 (UniRef100_E0A235), which is annotated as a “drought resistance protein” and was initially identified *via* transcriptomic studies in response to drought stress, and the researchers confirmed this gene’s role in abiotic stress tolerance *via* transgenic overexpression, which conferred increased tolerance for several abiotic stresses (Song et al., 2013).

Within the qTR_Gm12_1 QTL region (Gm12:35946078-36388059; Table 7 and Supplementary Table 1), we identified eight candidate genes based on GO annotation (Supplementary Table 1). Unfortunately, none of the candidate genes has any direct evidence for gene function, with all annotations inferred based on BLAST identification of presumably homologous genes from other species. Four of the candidate genes in this region are putative transcription factors (Glyma.12G199100, Glyma.12G199200, Glyma.12G199600, and Glyma.12G203100) whose annotations are associated with abiotic stress responses. Another interesting candidate gene is a homolog of a stomatal patterning gene (Glyma.12G202700). The qTR_Gm12_1 QTL displayed significant allelic differences for TR using H_2_O (Table 6), but no significant differences between parental alleles in the presence of the aquaporin inhibitor (TRAgNO_3_). Although, no obvious aquaporin gene is present within the region identified in the “Williams 82” reference genome, it remains possible that a nonobvious aquaporin-encoding gene (but not identified as such either due to problems with the assembly or diverged between “Williams 82” and the parental lines) is present within the qTR_Gm12_1 QTL region, which was affected by the silver inhibitor and forms the underlying genetic basis for qTR_Gm12_1 QTL.

Within the qLT_Gm12_1 QTL region associated with differential LT (Gm12:6971475.11867391, Table 7 and Supplementary Table 1), we identified a total of 312 genes, of which 29 were termed candidate genes based on GO term and KOG annotations. Only one gene was noted to have any functional characterization; Glyma.12G104800 was identified through RNA-Seq/RT-PCR analysis as associated with response to salt stress (Sun et al., 2019). Other potential candidate genes for the QTL include an aquaporin (Glyma.12G097800); eight genes are involved in abscisic acid biosynthesis/signaling (Glyma.12G087200, Glyma.12G089200, Glyma.12G094800, Glyma.12G096100, Glyma.12G098900, Glyma.12G103100, Glyma.12G106400, and Glyma.12G108900), and a there were large number of transcription factors with annotations, which suggest involvement in abiotic stress responses (Glyma.12G087000, Glyma.12G094500, Glyma.12G094800, Glyma.12G096100, Glyma.12G098800, Glyma.12G100100, Glyma.12G100600, Glyma.12G103100, Glyma.12G104500, Glyma.12G104600, Glyma.12G104800, Glyma.12G105400, Glyma.12G105600, Glyma.12G106400, and Glyma.12G110400).

## Discussion

### Phenotyping and Phenotypic Traits

Treating the soybean plants with AgNO_3_, aquaporin inhibitor reduced the TR responses (Table 3 and Figure 1). Silver inhibition has been linked to the sulfhydryl group of cysteine residue of AQPs resulting in blockage of the pore for water passage (Niemietz and Tyerman, 2002). A couple of studies using aquaporin inhibitor, silver nitrate on derooted, and intact soybean plants indicated that most soybean genotypes were quite sensitive to the treatment with silver nitrate (Sadok and Sinclair, 2010; Devi et al., 2015). However, they reported a varying range of DTRs across genotypes. In the current research, the DTR amount varied across genotypes, ranging from 0.46 to 74.6% (Supplementary Figure 1). Twelve RILs had very low DTR (#162, #89, #97, #74, #16, #31, #161, #30, #168, #71, #26, and #137) ranging from 0–5%, whereas the other eleven genotypes had DTR ranging from 5–10% (#52, #64, #17, #157, #91, #23, #34, #136, #73, #45, and #57) (Supplementary Figure 1). These genotypes were almost insensitive to AgNO_3_ exposure and can be categorized as slow wilting (Sadok and Sinclair, 2010).

The results also indicated that AgNO_3_ could be an effective way for high-throughput screening of drought tolerant soybean genotypes. Previous studies have suggested that silver nitrate (AgNO_3_) may be an effective initial screen for the expression of the TR_lim_ trait in a multitier screening system (Sadok and Sinclair, 2010; Choudhary and Sinclair, 2014; Shekoofa and Sinclair, 2018). In this work, the second tier (first being AgNO_3_ treatment) would involve selection of fewer genotypes for intensive and direct phenotyping for the TR response under increasing VPD conditions. Therefore, it allowed for considering not only the DTR trait, but also several other secondary traits to represent the reduction in TR response. A simple but less accurate screen that allows a large number of genotypes to be examined is a first-tier screen, followed by tiers of more sophisticated screens of decreasing numbers of genotypes (Sinclair, 2011).

Of all primary and secondary traits, those subjected to PCA, RDTR_K_, and RNDTR_K_ had the best correlation with LT (Figure 2). The regression curve of RDTRK and RNDTR_K_ with the LT shows that the slope is negligible for temperature rates below 32°C, but it drastically changes with increasing the temperature above 32°C (Figure 3). At optimum LT (24–32°C), the TR response was almost constant. These findings were confirmed through the direct measurement of TR responses under high VPD conditions at 32 and 37°C (i.e., the second-tier screening). Almost 88% of slow wilting genotypes (i.e., the least sensitive genotypes to AgNO_3_ exposure), 50% of moderate wilting genotypes, and 11% of high wilting genotypes (i.e., highly sensitive genotypes to AgNO_3_ exposure) expressed the TR_lim_ trait at 32°C. This was consistent with our hypothesis and previous studies done on other legume crops (Devi et al., 2010; Shekoofa et al., 2017). The genotypes with low sensitivity to aquaporin inhibitor, AgNO_3_, had the TR_lim_ trait with VPD breakpoint (BP) at about 1.3–2.9 kPa (Table 5). The TR_lim_ at lower VPD rates (i.e., 1.3–2.9 kPa) allows those genotypes to conserve moisture so that there would be more water available in the soil during late-season drought to sustain soybeans physiological activities and complete seed fill. The plants with TR_lim_ trait have also shown to delay wilting under rainfed conditions (Devi et al., 2010; Shekoofa et al., 2020).

Although the TR response results observed at 37°C (i.e., Experiment II) show that observations of the TR_lim_ trait at 32°C do not necessarily predict expression of the trait at higher temperatures, the expression of TR_lim_ trait reduced to about 43, 10, and 0% for slow, moderate, and high wilting genotypes from Experiment I, respectively. This could be because the rest of genotypes which expressed the TR_lim_ (i.e., VPD BP) at 32°C started to increase TR under 37°C to disperse excess temperature and heat. Shekoofa et al. (2016) reported that if very high temperatures are frequent, even to the point of being high enough to threaten plants with heat stress, it may be advantageous to consider corn hybrids that lose the TR_lim_ trait at 38°C rather than at 32°C or other temperatures below 38°C. However, soybean genotypes #16, #23, #73, and #87 allowed full expression of the TR_lim_ trait under both temperatures (i.e., 32 and 37°C) and displayed the maximum water conservation, no matter what the temperature regime was and performed better than both parents. Further evaluation of these genotypes should be done in field by simulating drought conditions.

### Genotyping and Quantitative Trait Locus Discovery

Quantitative trait loci analysis for the RILs population was done to associate the potential QTLs with the drought tolerance traits. Two QTLs (qTR_Gm10_1 and qTR_Gm12_1; Table 6) overlapped with two previously identified QTLs (qSV_Gm10 and qSV_Gm12) associated with drought responses from PI 416937 (Carpentieri-Pipolo et al., 2012). QTL conditioning the sensitivity of plants to silver nitrate were located in the same chromosomal regions reported by Carpentieri-Pipolo et al. (2012). Although, from investigations of pedigrees, it does not appear that the population (“Jackson” × “KS4895”) studied in the current research has any direct pedigree connection to the previously studied PI416937, but these QTL may be evidence that variation in the same underlying genes is causative.

Furthermore, it has been hypothesized that insensitivity to AgNO_3_ could be related to QTLs and the trait could be heritable (Devi et al., 2016). Our study also found that all primary and secondary traits had high broad sense heritability (H^2^ from 0.68 to 0.90) (Table 8). Therefore, these drought-tolerant traits can be used for phenotypic selection of soybean genotypes in future breeding efforts.

**TABLE 8:** Broad sense heritability (H^2^) values of all direct and indirect traits measured in this study.

Trait	H^2^
TR H_2_O	0.86
TR AgNO_3_	0.90
DTR	0.78
NDTR	0.88
RTR	0.70
RDTR_J_	0.77
RNDTR_J_	0.74
RRTR_J_	0.69
RDTR_K_	0.74
RNDTR_K_	0.87
RRTR_K_	0.68
LT (°C)	0.90

*H^2^ values range from 0 to 1; values closer to 1 have better heritability.*

The “KS4895” allele of the qTR_Gm10_1 QTL was associated with lower TR (Table 6). This was true for the H_2_O treatment (−0.63 mg H_2_O m2^–1^ s^–1^) and the AgNO_3_ treatment (−0.35 mg H_2_O m2^–1^ s^–1^) as well as the differential trait (TR H_2_O – AgNO_3_; −0.28 mg H_2_O m2^–1^ s^–1^). In contrast, the “KS4895” allele of the qTR_Gm12_1 QTL had higher TR under the H_2_O treatment (+0.27 mg H_2_O m2^–1^ s^–1^), and the differential trait (+0.25 mg H_2_O m2^–1^ s^–1^), but not with the AgNO_3_ treatment alone (Table 6).

We identified a relatively small number of candidate genes from within detected QTL regions (8, 8, and 29 for qTR_Gm10_1, qTR_Gm12_1, and qLT_Gm12_1, respectively) based on GO terms associated with abscisic acid, water transport, root development, leaf senescence, jasmonic acid, stomata, and/or salicylic acid. These candidate genes will be useful for future efforts to determine the causative genetic basis for the three QTLs we identified.

### Limitations of Our Study and Future Directions

Although one of the RIL parental lines (‘‘Jackson’’) has resequencing data publicly available,^3^ the other parental line (“KS4895”) to our knowledge has not been resequenced. Moreover, no resources for fine-level genetic mapping (e.g., near-isogenic lines) have previously been developed for detected QTL regions identified for the TR_lim_ traits. As such, fine-mapping, identification of gene polymorphisms, correlation of polymorphisms with water use efficiency, and TR_lim_ traits, and the ultimate cloning of causative polymorphisms from within QTL regions remains to future work.

Nevertheless, our results strongly suggest that advanced mapping populations can reveal QTLs for drought-tolerance traits under complicated genetic control to enhance the TR_lim_ trait (i.e., ability to tolerate late-season drought) in a RIL soybean population from crossing “KS4895” × “Jackson.” Validation of identified QTLs will be useful in molecular breeding of these favorable and informative QTL alleles for a superior cultivar with the ability to produce stable yield under water limiting conditions. It will be an interesting approach to associate these alleles with other agronomic traits, which are tightly linked to desirable drought tolerance traits to increase soybean yield and production under dryland conditions.

## Data Availability Statement

The datasets presented in this study can be found in online repositories. The names of the repository/repositories and accession number(s) can be found in the article/Supplementary Material.

## Author Contributions

AS and SS: conceptualization and writing – original draft preparation. AS, SS, and JG: methodology and data curation. AS: investigation, resources, visualization, supervision, and funding acquisition. AS, SS, JG, and AM: writing – review and editing. All authors have read and agreed to the published version of the manuscript.

## Conflict of Interest

The authors declare that the research was conducted in the absence of any commercial or financial relationships that could be construed as a potential conflict of interest.

## Publisher’s Note

All claims expressed in this article are solely those of the authors and do not necessarily represent those of their affiliated organizations, or those of the publisher, the editors and the reviewers. Any product that may be evaluated in this article, or claim that may be made by its manufacturer, is not guaranteed or endorsed by the publisher.

## Footnotes

1
https://github.com/kbroman/qtl/blob/master/R/droponemarker.R

2 www.soybase.org, accessed November 11, 2021.

3 https://soybase.org/projects/SoyBase.B2014.02.php, accessed November 17, 2021.

## Supplementary Material

Supplementary Figure 1 Variation of decrease in transpiration rate (DTR) and normalized decrease in transpiration rate (NDTR) among 122 soybean genotypes.

Supplementary Figure 2 Diagram of a two-tiered screening method to identify soybean genotypes with the TR_lim_ trait.

Supplementary Table 1 Candidate genes from GO term analysis from within QTL regions identified in this study.

Supplementary File 1 Heritability analysis and values of all direct and indirect traits.

Supplementary File 2 Details on genetic map and marker distribution.

Supplementary File 3 R/QTL package file containing genotypic and phenotypic data used for genetic mapping.

## Abbreviations

TR_lim_: limited transpiration
VPD: vapor pressure deficit
RIL: recombinant inbred line
QTL: quantitative trait locus
TR: transpiration rate
DTR: decrease in transpiration rate
LT: leaf temperature
AQPs: aquaporins
TRH_2_O: transpiration in water
TRAgNO_3_: transpiration rate following exposure to silver nitrate
PCA: principle component analysis
H^2^: broad sense heritability
DAP: days after planting
BP: breakpoint.
