# Supplementary figures and images for: Phenotyping and Quantitative Trait Locus Analysis for the Limited Transpiration Trait in an Upper-Mid South Soybean Recombinant Inbred Line Population (“Jackson” × “KS4895”): High Throughput Aquaporin Inhibitor Screening
Source: Front Plant Sci. 2022 Jan 20;12:779834. doi: 10.3389/fpls.2021.779834 (PMC8811256; doi:10.3389/fpls.2021.779834)

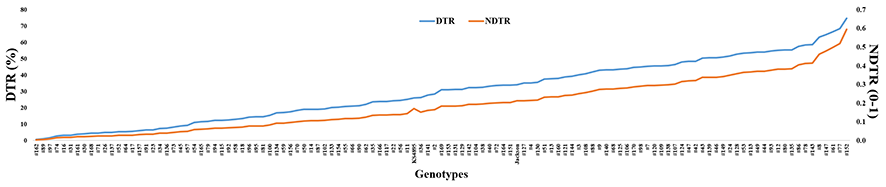

Supplement: Supplementary Figure 1 — Variation of decrease in transpiration rate (DTR) and normalized decrease in transpiration rate (NDTR) among 122 soybean genotypes. [file Image_1.TIF]

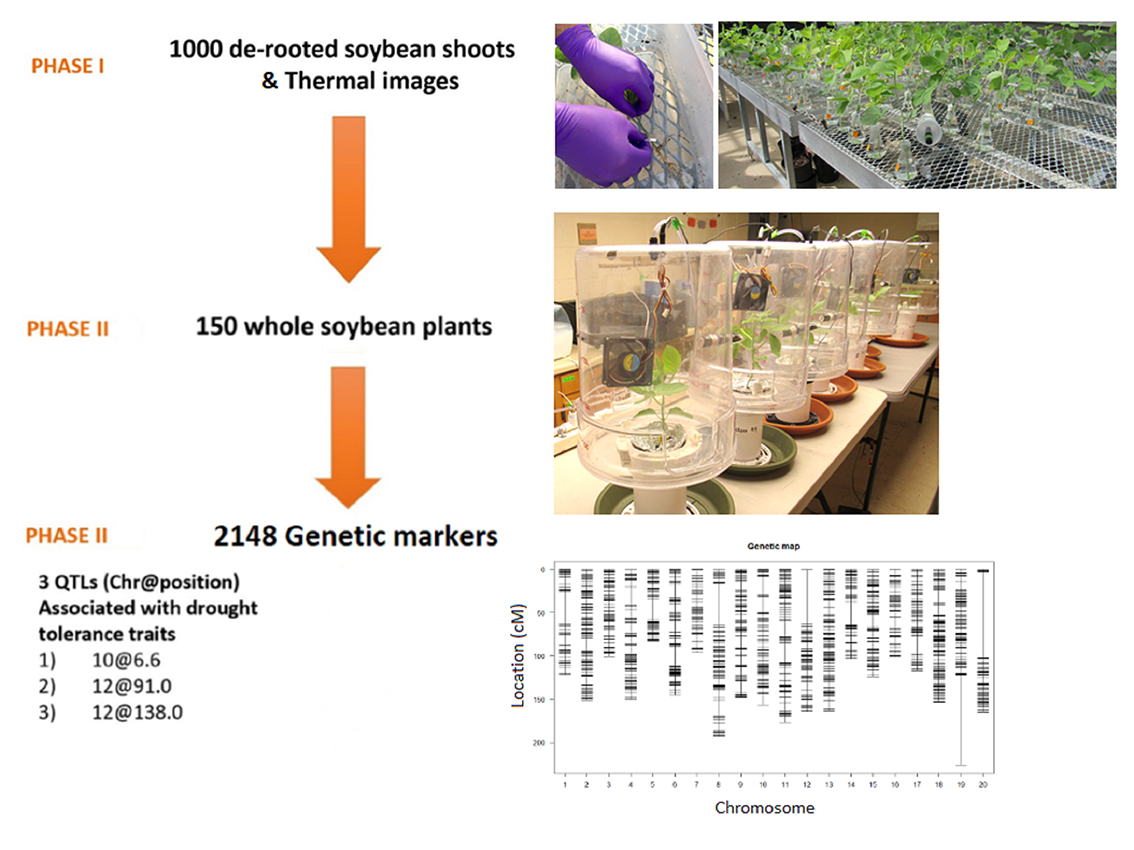

Supplement: Supplementary Figure 2 — Diagram of a two-tiered screening method to identify soybean genotypes with the TRlim trait. [file Image_2.TIF]
